# Unraveling the mechanisms of resistance to *Sclerotium rolfsii* in peanut (*Arachis hypogaea* L.) using comparative RNA-Seq analysis of resistant and susceptible genotypes

**DOI:** 10.1371/journal.pone.0236823

**Published:** 2020-08-03

**Authors:** Tejas C. Bosamia, Sneha M. Dodia, Gyan P. Mishra, Suhail Ahmad, Binal Joshi, Polavakkalipalayam P. Thirumalaisamy, Narendra Kumar, Arulthambi L. Rathnakumar, Chandramohan Sangh, Abhay Kumar, Radhakrishnan Thankappan

**Affiliations:** 1 ICAR-Directorate of Groundnut Research (ICAR-DGR), Junagadh, India; 2 Indian Agricultural Research Institute (IARI), New Delhi, India; 3 ICAR-National Research Centre on Litchi, Muzaffarpur, India; Nanjing Agricultural University, CHINA

## Abstract

Stem rot, a devastating fungal disease of peanut, is caused by *Sclerotium rolfsii*. RNA-sequencing approaches have been used to unravel the mechanisms of resistance to stem rot in peanut over the course of fungal infection in resistant (NRCG-CS85) and susceptible (TG37A) genotypes under control conditions and during the course of infection. Out of about 290 million reads, nearly 251 million (92.22%) high-quality reads were obtained and aligned to the *Arachis duranensis* and *Arachis ipaensis* genomes with the average mapping of 78.91% and 78.61%, respectively. In total, about 48.6% of genes were commonly regulated, while approximately 21.8% and 29.6% of uniquely regulated genes from *A*. *duranensis* and *A*. *ipaensis* genomes, respectively, were identified. Several annotated transcripts, such as receptor-like kinases, jasmonic acid pathway enzymes, and transcription factors (TFs), including WRKY, Zinc finger protein, and C2-H2 zinc finger, showed higher expression in resistant genotypes upon infection. These transcripts have a known role in channelizing the downstream of pathogen perception. The higher expression of WRKY transcripts might have induced the systemic acquired resistance (SAR) by the activation of the jasmonic acid defense signaling pathway. Furthermore, a set of 30 transcripts involved in the defense mechanisms were validated with quantitative real-time PCR. This study suggested PAMP-triggered immunity as a probable mechanism of resistance, while the jasmonic acid signaling pathway was identified as a possible defense mechanism in peanut. The information generated is of immense importance in developing more effective ways to combat the stem rot disease in peanut.

## Introduction

Groundnut or peanut (*Arachis hypogaea* L.) is an allotetraploid crop (2n = 4x = 40, AABB genome), which is cultivated in several parts of the world, mainly by small and marginal farmers under low-input conditions [1, 2]. It is a major oilseed crop that has been grown in an area of 27.94 million ha worldwide, with an average productivity of 1685 kg/ha [3]. India ranks first in the cultivation of peanut with 5.3 million ha of the world’s land area and second in production (9.17 million tons), with the average productivity of 1731 kg/ha [3]. In peanut, several kinds of biotic stresses not only limit the overall crop productivity but also affect seed quality. Stem rot disease is caused by a necrotrophic, soil-borne fungal pathogen *Sclerotium rolfsii* Sacc. (*Athelia rolfsii*), which may incur yield losses of 10–40%, especially under irrigated conditions [4, 5]. Moreover, the oxalic acid produced by *S*. *rolfsii* causes a blue discoloration on the seed surface and ultimately affects the overall seed quality [6].

*S*. *rolfsii* has an extensive host range, and it is challenging to eliminate it from the infested soil [7]. The initial symptom of an infection in the plant includes the dark-brown lesions on the stem at or just beneath the soil surface, followed by the progressive yellowing and wilting of leaves [8]. The management of stem rot disease is quite complex, as the fungus forms sclerotia, which can survive for an extended period in soil due to the presence of melanin in the outer membrane [9]. In order to manage the stem rot infection, the development of resistant varieties is considered a more economical and environmentally friendly approach compared to the fungicide application. Although no peanut genotype has yet been reported to be resistant to *Sclerotium rolfsii* infection, some genotypes have field resistance [5, 10, 11].

Moreover, the molecular basis of resistance to *Sclerotium rolfsii* in peanut is very poorly understood. Host plant resistance is used as a fundamental component of integrated disease management, as it offers early protection against disease development and simultaneously reduces pesticide use [5]. An inclusive understanding of host-pathogen interactions requires information about gene expression changes in both the pathogen and the host. Thus, it is essential to gather comprehensive information about the genes involved in host-pathogen interaction so that the novel genes can be identified and used in stem rot resistance breeding programs.

Transcriptome sequencing or RNA-Seq is a robust and cost-effective technology, providing deeper coverage and high-resolution profiling of transcripts [12]. This technique is widely used for understanding host-pathogen interaction as well as elucidating plant defense pathways [13, 14]. Moreover, it has been successfully used to unfold various developmental pathways [15], oil accumulation pathways [16], and in response to biotic [17] and abiotic stimuli in peanut [18, 19]. An RNA-Seq transcriptome analysis of *Brassica* infected with *Sclerotinia sclerotiorum* revealed that the genes involved in pathogen recognition, MAPK signaling cascade, regulation of WRKY transcription factors, and jasmonic acid and ethylene signaling pathways, impart resistance [20, 21]. Furthermore, Jogi et al. [22] have also identified a set of genes possibly involved in response to the pathogen. However, the limited information generated from a few studies cannot provide a comprehensive understanding of the defense response of peanut plants to the stem rot.

Genome sequencing of the peanut progenitors, *A*. *duranensis* and *A*. *ipaensis*, along with the next-generation sequencing platform like Illumina, provide new prospects to monitoring the defense response against *S*. *rolfsii* in peanut. Therefore, this study aimed at understanding the resistance reaction at the transcriptomic level in peanut infected with *S*. *rolfsii*.

## Materials and methods

### Plant material and disease induction

The highly virulent strain of *Sclerotium rolfsii* (*DGR-SR-8*) was isolated from naturally infected peanut plant at ICAR-Directorate of Groundnut research (DGR), Junagadh, Gujarat using the tissue segment method on potato dextrose agar (PDA) medium [23]. The pure culture was then obtained by the hyphal tip method, maintained on PDA, grown and multiplied under aseptic conditions on autoclaved sorghum grains for 15 days [11].

A stem rot-resistant genotype, NRCG-CS85 [24] and a susceptible genotype, TG37A were used for the RNA-Seq analysis. The genotypes NRCG-CS85 and TG37A were derived from the cross between [(CT 7–1 × SB11) × *A*. *kretschmeri*] and (TG 25 × TG 26), respectively. The seeds from these genotypes were soaked in water and sown in earthen pots (one seed per pot) containing sterilized vermiculite. Plants were then grown in a glasshouse at 28°C and 14 h photoperiod. Wheat straw was spread around the base of the potted plants. Plants (70 days old) were taken for their reaction against *S*. *rolfsii*, and the infected sorghum grain inoculums were applied near the main stem on each plant (2 g per pot) (S1 Fig). The control plants were only given distilled water. In the glasshouse, the temperature and humidity were maintained at 28°C and 70–80%, respectively, most favorable for the pathogen multiplication, leading to the infection [5, 25].

Twelve days after inoculation, the stem was cut, immersed in RNAlater solution (Sigma), and then stored at −70°C. A total of four samples, including resistant (NRCG-CS85) and susceptible (TG37A) genotypes of plants inoculated with *S*. *rolfsii* and their respective controls, were collected. Three biological replicates were considered for each sample. Furthermore, stem samples from each replicate were pooled for RNA-Seq analysis to improve the detection accuracy.

### RNA extraction, library construction, and RNA-sequencing

Total RNA was extracted from the frozen stem samples using the RNeasy plant mini kit with an on-column DNase digestion (Qiagen, Hilden, Germany) according to the manufacturer’s instructions. RNA quality was assessed on a 2% agarose gel, and the RNA integrity number (RIN) was measured using the Bioanalyzer 2100 RNA 6000 Nano Kit (Agilent Technologies, Santa Clara, CA, USA). RNA samples with 260/280 ratio of 2.0–2.1, 260/230 ratio of 2.0–2.3, and RIN value of > 7.0 were used for the construction of cDNA library using TruSeq mRNA Library Prep kit (Illumina Inc., USA). cDNA libraries and both ends of the inserts were then sequenced using the Illumina HiSeq 2500 platform (Illumina Inc., USA) at Sci-Genomics Labs Pvt. Ltd, Kerala, India.

### Sequence data pre-processing and rRNA removal

The quality of raw sequencing data was assessed using open-source software, FastQC (www.bioinformatics.babraham.ac.uk/projects/fastqc), and then the adapter sequences, low-quality bases (Phred score of < 30), and short sequences (read length of < 50 bp), were trimmed using AdapterRemoval-v2 (version 2.2.0) and in-house scripts. The resulting high-quality reads were trimmed, and the size-selected libraries were subjected to ribosomal RNA (rRNA) removal procedure. The reads were then aligned against the Silva database using Bowtie2 (version 2.2.9), Sam-tools (version 0.1.19), Sambamba (version 0.6.5), and BamUtil (version 1.0.13) tools. Moreover, the in-house scripts were deployed for a workflow process.

### Read alignment

The pre-processed, high-quality reads constituting removed rRNA were aligned to the assembly of chromosomal pseudomolecules of *Arachis duranensis* and *Arachis ipaensis*, the diploid ancestors of cultivated groundnut (PeanutBase.org). The alignment was performed using the TopHat V2.0.13 (default parameters), and the resulting alignment (BAM file format) was used to generate transcript annotations in GTF format using Cufflinks V 2.2.1 with default parameters.

### Differential gene expression analysis

After aligning the reads to the reference genome, the read counts were normalized by calculating the fragments per kilobase of exon model per million reads mapped (FPKM) of each gene, and the expression per gene/transcript was estimated using Cufflinks program (version 2.2.1). The differential expression analysis was performed using Cuffdiff, a part of the Cufflinks package with default parameters. The differentially expressed transcripts for each of the four samples were identified based on a P-value (< 0.05) and log2 fold change (log2FC) ≥ 2 for up-regulated genes or log2FC ≤ –2 for down-regulated genes for further analysis. The transcripts were functionally classified and presented in a heat map using Multi Experiment Viewer (MeV) v4.9.0, and the number of transcripts among and between the conditions was plotted as a Venn diagram using Vennplex [26].

### Gene ontology (GO) enrichment analysis and pathway analysis of transcripts

The GO terms for all four comparisons and graphically expressed genes were identified by transcript annotation using the online WEGO tool [27]. The identified GO terms were enriched using an online tool PlantRegMap [28] (http://plantregmap.cbi.pku.edu.cn) at P ≤ 0.01. Enriched terms were then visualized as a scatter plot using Revigo [29] (http://revigo.irb.h;). A web-based server, namely KAAS (KEGG (Kyoto Encyclopedia of Genes and Genomes) Automatic Annotation Server), was used for ortholog assignment and mapping of the CDs to the biological pathways.

### Validation of the expression profiles by qPCR

In order to study the initial response of the plant to the pathogen, the stem tissues of both control and treated genotypes were harvested at 0, 24, 48, 72, 96 h, and 12 d after inoculation. The samples harvested at 12 days after inoculation were used to compare RNA-Seq data with qPCR data. A total of 49 disease resistance-responsive genes were chosen based on their function to validate the expression pattern revealed by RNA-Seq data using qPCR analysis. The primers were designed using the Batch Primer3 software [30]. The cDNA sample diluted 1:10 in nuclease-free water was prepared before conducting qPCR analysis.

The qPCR was performed using 2×SYBR® Green ROX qPCR FAST mastermix (QIAGEN, USA) on a 48-well StepOne™ Real-Time PCR System (Applied Biosystems), and the PCR amplification was performed using a 3-step program; 10 min at 95°C, then 40 cycles of 95°C for 15 sec, and 60°C for 30 sec. The stability of endogenous reference genes during the course of stem rot disease was analyzed using the program RefFinder [31]. To normalize the variance between the samples, beta-actin was used as an endogenous control for gene expression analysis, and 2^–ΔΔCT^ (Livak) method [32] was used for data analysis.

## Results

### RNA-Seq mapping and statistical analysis

RNA-Seq generated a total of 290 million reads from all four samples, with an average GC content of 46.09%. After passing the quality check and removing the low complexity reads, adaptor/primer sequences, and rRNA sequences with about 251 million (86.48%) high-quality reads (HQRs) and Phred value of ≥ 30, were obtained (Table 1). Raw sequencing reads were deposited in the NCBI Sequence Read Archive (SRA, http://www.ncbi.nlm.nih.gov/sra) under the accession number PRJNA521728. The HQRs were aligned to the genomes of *Arachis duranensis* and *Arachis ipaensis* using the top head alignment tool (Table 2). The reads mapped to the *A*. *duranensis* and *A*. *ipaensis* reference genomes showed the range of 63.83% to 87.61% (an average of 78.91%), and 64.56% to 86.64% (an average of 78.61%), respectively, per library. Moreover, HQRs were also aligned to the genome of *Athelia rolfsii* to investigate the fungal transcripts transferred through the host plant. A total of 549 and 168 reads of RI and SI samples, respectively, were mapped to the *Athelia rolfsii* genome.

**Table 1:** Statistics of transcriptome sequencing in resistant and susceptible genotypic combinations under infected and control conditions.

Sample Name	RC	RI	SC	SI	Total
No. of raw reads (Total read counts)	71,977,040	76,316,072	79,092,064	63,449,258	290,834,434
No. of bases (Gb)	7.20	7.63	7.91	6.34	29.08
GC (%)	42.48	47.96	46.09	47.84	46.09
≥Q30 (%)	93.33	91.41	92.42	91.73	92.22
QC failed (%)	4092082 (5.69)	10275636 (13.46)	13552346 (17.13)	11388770 (17.95)	39308834 (13.52)
High quality reads (%)	67884958 (94.31)	66040436 (86.54)	65539718 (82.87)	52060488 (82.05)	251525600 (86.48)

Where RC: Resistant control; RI: Resistant inoculated; SC: Susceptible control; SI: Susceptible inoculated.

**Table 2:** Mapping statistics of HQRs to *A*. *duranensis* and *A*. *ipaensis* genomes.

Mapping details	RC	RI	SC	SI	Total
**Mapping to *A*. *duranensis***					
Aligned reads count (%)	59471599 (87.61)	55427054 (83.93)	41837077 (63.83)	41751818 (80.2)	198487548 (78.91)
Unaligned reads count (%)	8413359 (12.39)	10613382 (16.07)	23702641 (36.17)	10308670 (19.8)	53038052 (21.09)
**Mapping to *A*. *ipaensis***					
Aligned reads count (%)	58813016 (86.64)	54944981 (83.2)	42310671 (64.56)	41651365 (80.01)	197720033 (78.61)
Unaligned reads count (%)	9071942 (13.36)	11095455 (16.8)	23229047 (35.44)	10409123 (19.99)	53805567 (21.39)
**Mapping to *Athelia rolfsii***					
Aligned reads count	NA	549	NA	168	717

### Identification of differentially expressed genes

A detailed four-way comparison of each sample was performed with both the *Arachis duranensis* and *Arachis ipaensis* genomes to identify the number of up-regulated and down-regulated transcripts at p ≤ 0.05 and log2 fold change ≤ ±2.0 (Table 3). The mapping to the *A*. *duranensis* genome revealed a total of 1772, 107, 821, and 1260 up-regulated genes, while 1125, 220, 1407, and 1105 genes were down-regulated in RI_RC, SI_SC, RC_SC, and RI_SI combinations, respectively. Similarly, mapping to the *A*. *ipaensis* genome revealed 1768, 104, 831, and 1272 up-regulated genes, while 1145, 251, 1458, and 1160 genes were down-regulated in RI_RC, SI_SC, RC_SC, and RI_SI combinations, respectively (Table 3). Thus, more up-regulated transcripts were found in RI_RC and RI_SI combinations; however, more down-regulated transcripts were observed in SI_SC and RC_SC combinations.

**Table 3:** Comparison of transcripts in various sample combinations.

Sample Comparisons	*A*. *duranensis*		*A*. *ipaensis*	
	Up regulated p-value ≤0.05 (0.01)	Down regulated p-value ≤0.05 (0.01)	Up regulated p-value ≤0.05 (0.01)	Down regulated p-value ≤0.05 (0.01)
RI_RC	1772 (880)	1125 (511)	1768 (855)	1145 (513)
SI_SC	107 (43)	220 (57)	104 (39)	251 (75)
RC_SC	821 (258)	1407 (675)	831 (334)	1458 (677)
RI_SI	1260 (331)	1105 (554)	1272 (642)	1160 (574)

The values in parenthesis are transcripts having a p-value ≤0.01

In order to estimate the proportion of transcripts from *A*. *duranensis* and *A*. *ipaensis* genomes, all four comparisons were performed using both the genomes. Venn diagram showed 2261 (48.6%) common transcripts, while 1012 (21.8%) and 1378 (29.6%) uniquely regulated transcripts were generated from *A*. *duranensis* and *A*. *ipaensis* genomes, respectively (S2 Fig). Dodia et al. [5] reported the identified stem rot resistance quantitative trait loci (QTLs) and candidate genes of the *A*. *ipaensis* genome. Since the total number of transcripts mapped to the *A*. *ipaensis* genome was more than that of the *A*. *duranensis* genome, the *A*. *ipaensis* genome was selected for further in-depth analysis.

### Classification of differentially expressed transcripts

**Fig 1:**
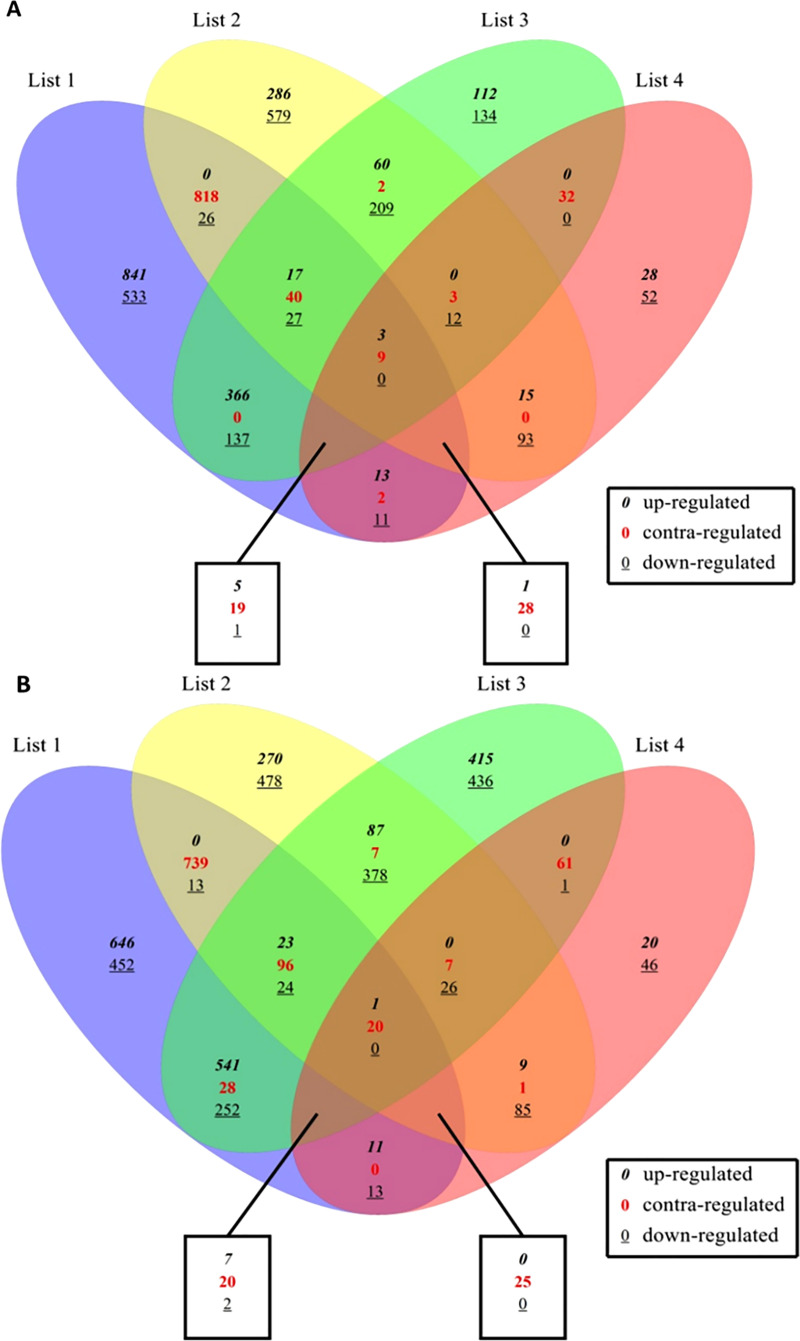
Venn diagram representing the number of transcripts as obtained by mapping with (A). *Arachis duranensis* genome, and (B). *Arachis ipeansis* genome of resistant and susceptible peanut genotypes upon *Sclerotium rolfsii* infection. Where List 1: RI_RC; List 2: RC_SC; List 3: RI_SI; List 4: SI_SC.

The up-regulated, down-regulated, and contra-regulated transcripts for both the *A*. *duranensis* and *A*. *ipaensis* genomes were represented in the Venn diagram in four groups (Fig 1), which were further divided into 15 subgroups. A total of 4514 unique transcripts mapped to the *A*. *duranensis* genome were identified across all four comparisons. Among these transcripts, 30.44% (1374/4514), 1.77% (80/4514), 19.16% (865/4514), and 5.45% (246/4514) group-specific transcripts were found in RI_RC, SI_SC, RC_SC, and RI_SI combinations, respectively. For *A*. *ipaensis* genome, a total of 5238 unique transcripts were identified across all four comparisons. The identified group-specific transcripts were 20.96% (1098/5238), 1.26% (66/5238), 14.28% (748/5238), and 16.24% (851/5238) in RI_RC, SI_SC, RC_SC, and RI_SI combinations, respectively. Moreover, 26 and 24 commonly regulated transcripts were found across all four combinations in *A*. *duranensis* and *A*. *ipaensis* genomes, respectively.

### Gene ontology

**Fig 2:**
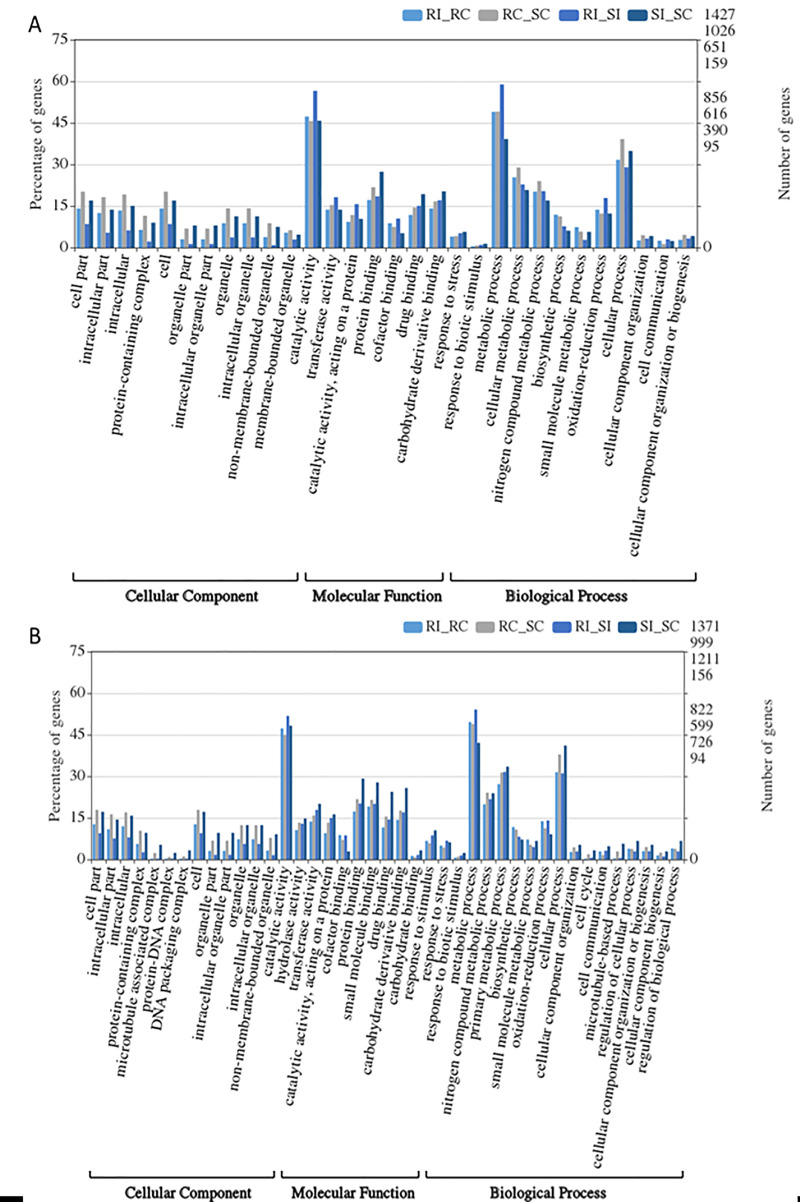
Identified gene ontology terms of DEGs by mapping to (A). *A*. *duranensis* genome and (B). *A*. *ipaensis* genome. These GO terms are classified into three categories (cellular component, molecular function, and biological processes).

A total of 1828/2913 (62.75%), 1332/2289 (58.19%), 1615/2432 (66.40%), and 209/355 (58.87%) transcripts were assigned to GO terms in RI_RC, RC_SC, RI_SI, and SI_SC combinations, respectively. In addition, out of 4984 transcripts assigned to 8922 GO terms, 3212, 2399, 2922, and 389 GO terms were assigned to the transcripts in RI_RC, RC_SC, RI_SI, and SI_SC combinations, respectively. Moreover, 3437, 1207, and 4278 transcripts were categorized into three main GO categories, including biological process, cellular component, and molecular function, respectively (Fig 2).

### GO enrichment analysis

**Fig 3:**
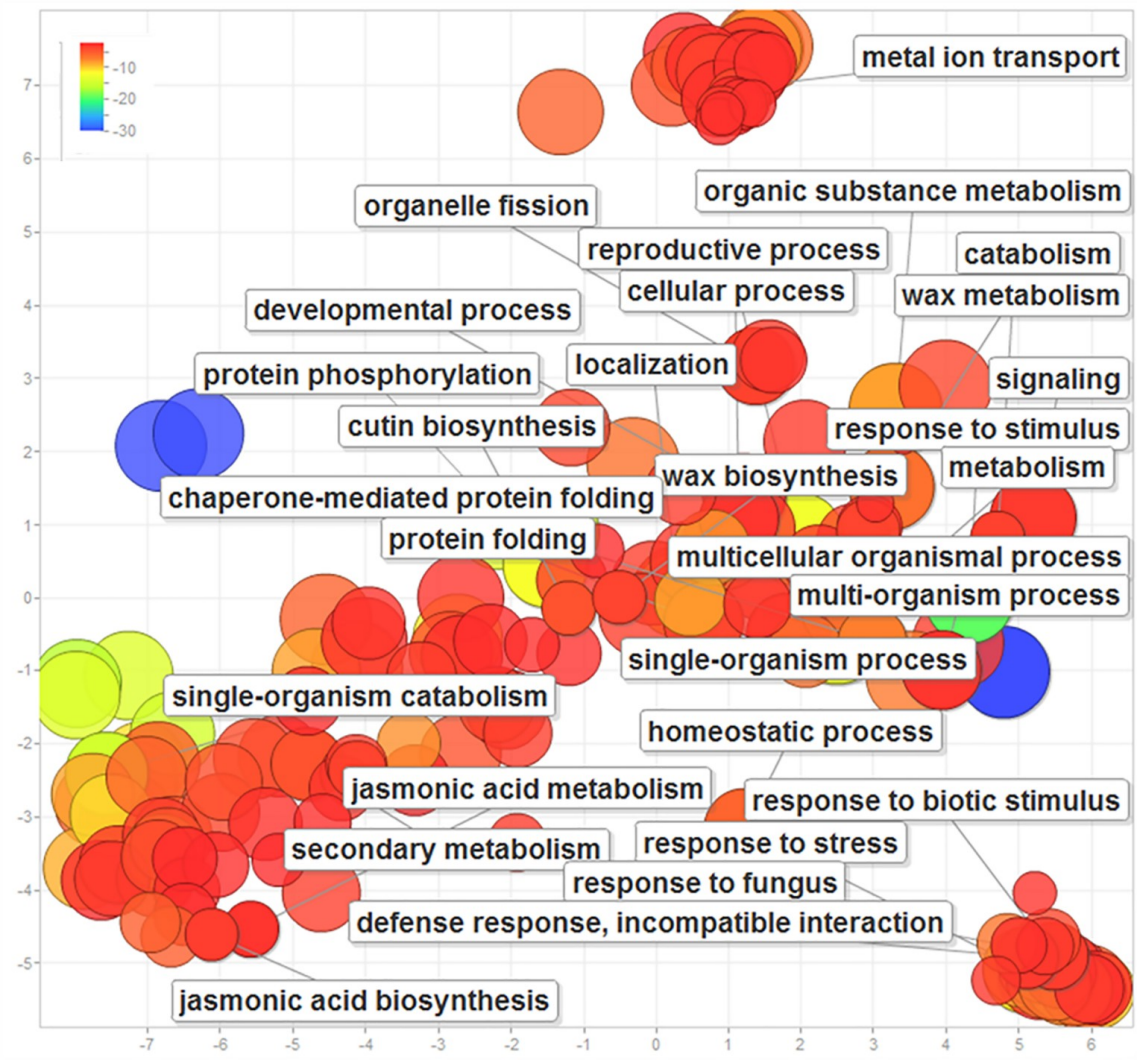
Scatter-plot showing over-represented GO term (P<0.01) in all comparisons with labels on the disease responsive terms. Different shades in circles indicate the difference in P-values (as given in the scale), whereas the bubble size indicates the frequency of GO term.

Of all GO terms assigned to the transcripts in four combinations of resistant and susceptible genotypes, 21.9% was enriched (Table 4). The GO molecular function and cellular process terms had the highest frequency. The majority of the enriched GO terms were found to be involved in various metabolic processes such as primary metabolic process, organic substance metabolic process, cellular metabolic process, nitrogen compound metabolic process, and biosynthetic process (Fig 3). The frequency of response to stimulus (GO:0050896) was 12.21%, while various disease-specific enriched terms such as response to biotic stimulus (GO:0009607); response to fungus (GO:0009620); defense response, incompatible interaction (GO:0009814); jasmonic acid metabolic process (GO:0009694); jasmonic acid biosynthetic process (GO:0009695); wax biosynthetic process (GO:0010025) and cutin biosynthetic process (GO:0010143), were also identified.

**Table 4:** Details of GO and GO enrichment in different resistant and susceptible combinations.

Treatment pair	Total number of transcripts	Number of transcripts assigned to GO term	Number of GO term assigned	Number of GO enrichment (%)
RI_RC	2913	1828	3212	685 (21.3)
RC_SC	2289	1332	2399	486 (20.2)
RI_SI	2432	1615	2922	536 (18.3)
SI_SC	355	209	389	250 (64.2)
**Total**	**7989**	**4984**	**8922**	**1957 (21.9)**

### Differential expression of candidate genes and their expression patterns during stem rot infection

**Fig 4:**
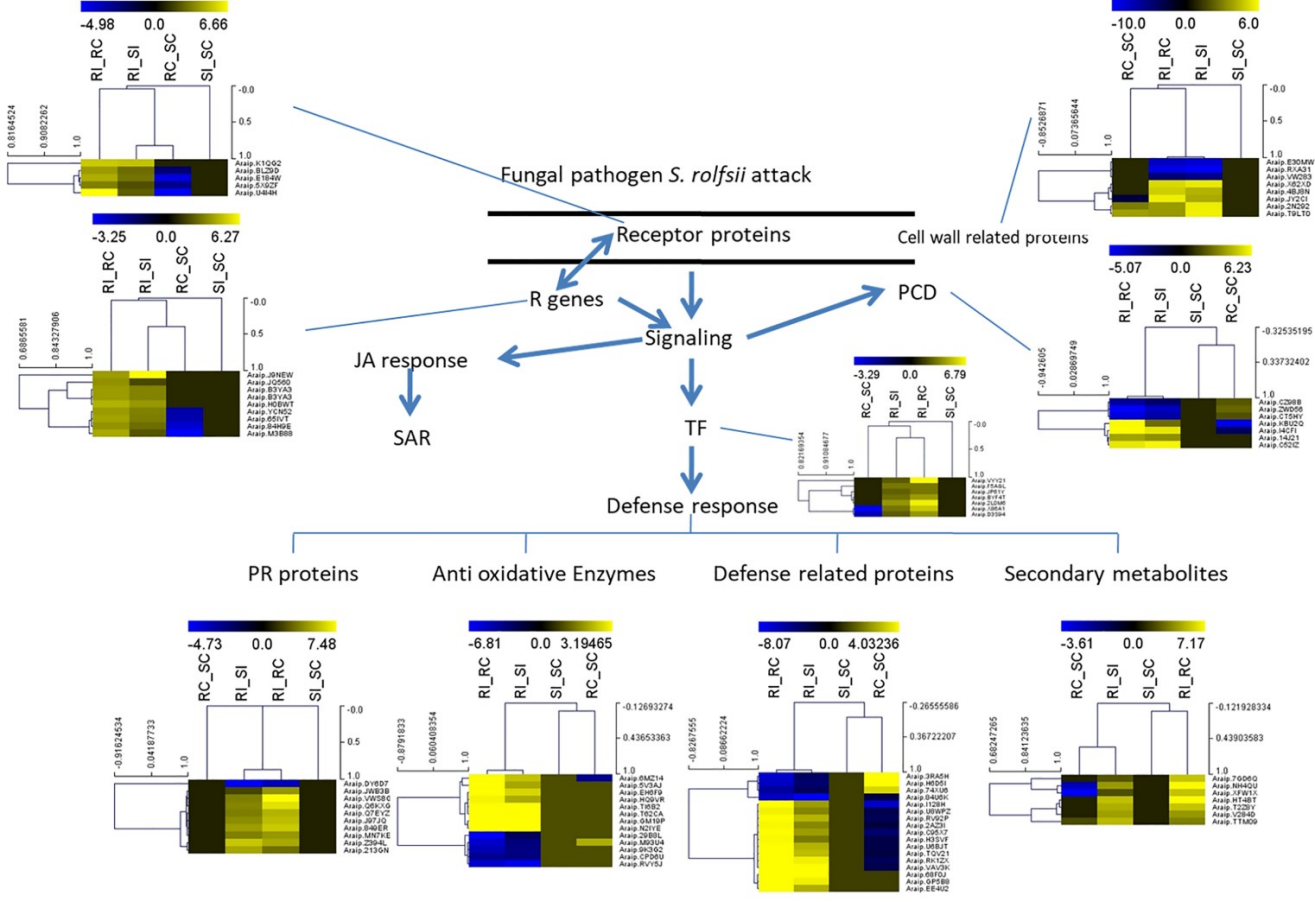
A putative defense response against the infection with *Sclerotium rolfsii* in peanut through various types of transcripts. The relative expression levels of each transcript are presented as a heat map (hierarchical clustering with Pearson’s uncentered correlation and complete linkage method). Scale represents down-regulation/low expression and up-regulation/high expression.

The expression patterns for all the transcripts were analyzed in four combinations, and several disease-responsive transcripts were identified based on their GO allotment in RI_RC sample comparison (Fig 4), as this combination was considered more important than the others. As a part of the defense mechanism, the fungal pathogen was recognized by the receptor proteins and resistance genes (R genes) in plants, leading to the downstream gene expression in the cascade. These defense responses were divided into different classes based on their functions.

Receptor-like kinases (RLKs) are the group of proteins involved in the recognition of fungi; these proteins include calmodulin binding protein and various classes of protein kinases, such as receptor-like serine/threonine protein kinase 2, protein kinase superfamily, and LRR receptor-like serine/threonine protein kinase. A total of 22 different types of RLKs were identified in the RI samples, found distributed at 63 different loci in the *A*. *ipaensis* genome. In the RI and SI comparison, 10 different types of RKLs from 32 loci were found stimulated. Interestingly, only six RLKs were found upregulated in RI when compared with SI, while rest of the RLKs showed down-regulation in RI when compared to SI (S1 Table). Among the kinases, the transcripts encoding various types of serine/threonine kinases were found to be differentially expressed mostly in the inoculated samples than the control samples.

The R gene-related transcripts included TIR-NBS-LRR proteins (TNLs), CC-NBS-LRR proteins (CNLs), LRR/NB-ARC domain-based proteins, dirigent-like proteins, putative LRR-containing proteins, cysteine-rich TM module stress tolerance proteins, and disease-resistance response protein. Moreover, a total of 34 different types of R gene-related transcripts were recorded in the RI samples, found distributed at 61 different loci in the *A*. *ipaensis* genome (S2 Table). Among these transcripts, the NBS-LRR class of R-genes was the most abundant, and it was also highly up-regulated in the RI compared to the RC and SI samples.

The differentially expressed transcription factors (TFs) included WRKY proteins, Zinc finger proteins, C2H2 zinc finger proteins, Ring-H2 finger protein, C3HC4-type zinc finger, bHLH, NAC, and MYB (Fig 4). Among these TFs, the zinc finger class was the most abundant, followed by the MYB class. Moreover, The C2H2 zinc finger protein 2, C3HC4-type zinc finger protein, and WRKY-type were highly up-regulated in the RI compared to the RC and SI samples.

Plants are known to synthesize pathogenesis-related proteins (PR-proteins), antioxidative enzymes, disease-responsive proteins, and various secondary metabolites in response to the fungal infection [33]. Eight classes of PR-proteins, including PR–1, PR–3, PR-thaumatin superfamily protein, defensin, thioredoxin, beta-glucosidase 43, peroxidase, and chitinase, found to be differentially expressed in RI and RC samples. Among these proteins, PR–1, PR-thaumatin superfamily protein, and defensin, were found to be highly up-regulated in the RI compared to the control sample. However, beta-glucosidase–43 protein was found to be down-regulated in the RI sample compared to the control sample.

During fungal infection, various classes of antioxidative enzymes, polyamine oxidase, peroxidase superfamily protein, the alpha/beta hydrolase superfamily protein, PAL–2, glutathione reductase, respiratory burst oxidase protein, L-ascorbate oxidase, and classes of lipoxygenase proteins, were found to be up-regulated in the RI compared to the RC. However, proteins that increase the production of free radicals such as oxygen-evolving enhancer protein, myo-inositol oxygenase, and GDSL-like Lipase/Acylhydrolase superfamily protein, were down-regulated in the RI sample compared to the control and the SI sample. In addition to antioxidative enzymes, the defense-responsive proteins/enzymes such as 4-coumarate: CoA ligase 3, caffeoyl-CoA 3-O-methyltransferase, arogenate dehydratase 6, and D-3-phosphoglycerate dehydrogenase, were up-regulated in the RI sample compared to the RC and SI samples. The genes responsible for the production of secondary metabolites, such as flavonoid/isoflavonoid, terpenoids, and isoprenoid, showed upregulation in the RI sample compared to the RC sample. However, plant cell wall proteins such as Exp3 and Exp4 were up-regulated in the RI sample compared to the RC sample (Fig 4). However, there was an upregulation of the plant cell wall-related enzymes such as pectin esterase inhibitor 7 and chitinase in the RI sample compared to the control sample.

### Potential transcripts underlying QTL for resistance to stem rot

In order to identify the candidate genes, the differentially expressed genes (DGEs) were searched for their position in the chromosomal regions corresponding to the confidence intervals of the QTLs determined in an earlier study [5]. Three main QTLs affecting the chromosomes B04, B06, and B10 were identified spanning 5.2, 7.5, and 33 Mb regions, respectively. The integration of genetic and physical positions led to the identification of 3, 16, and 5 transcripts across all four samples as positional disease-responsive candidate genes underlying QTL regions of the chromosomes B04, B06, and B10, respectively (S3 Table). These regions were comprised of disease-responsive genes such as receptor-like kinases and peroxidase superfamily protein on B04 chromosome. The protein kinases family, WRKY TF family, zinc finger protein, and lipoxygenase related protein were located on B06 chromosome. Whereas terpene synthase, CAP superfamily protein (PR–1), cinnamyl-alcohol dehydrogenase, and zinc-binding alcohol dehydrogenase family protein were positioned on B10 chromosome.

### Gene expression analysis by quantitative PCR

**Fig 5:**
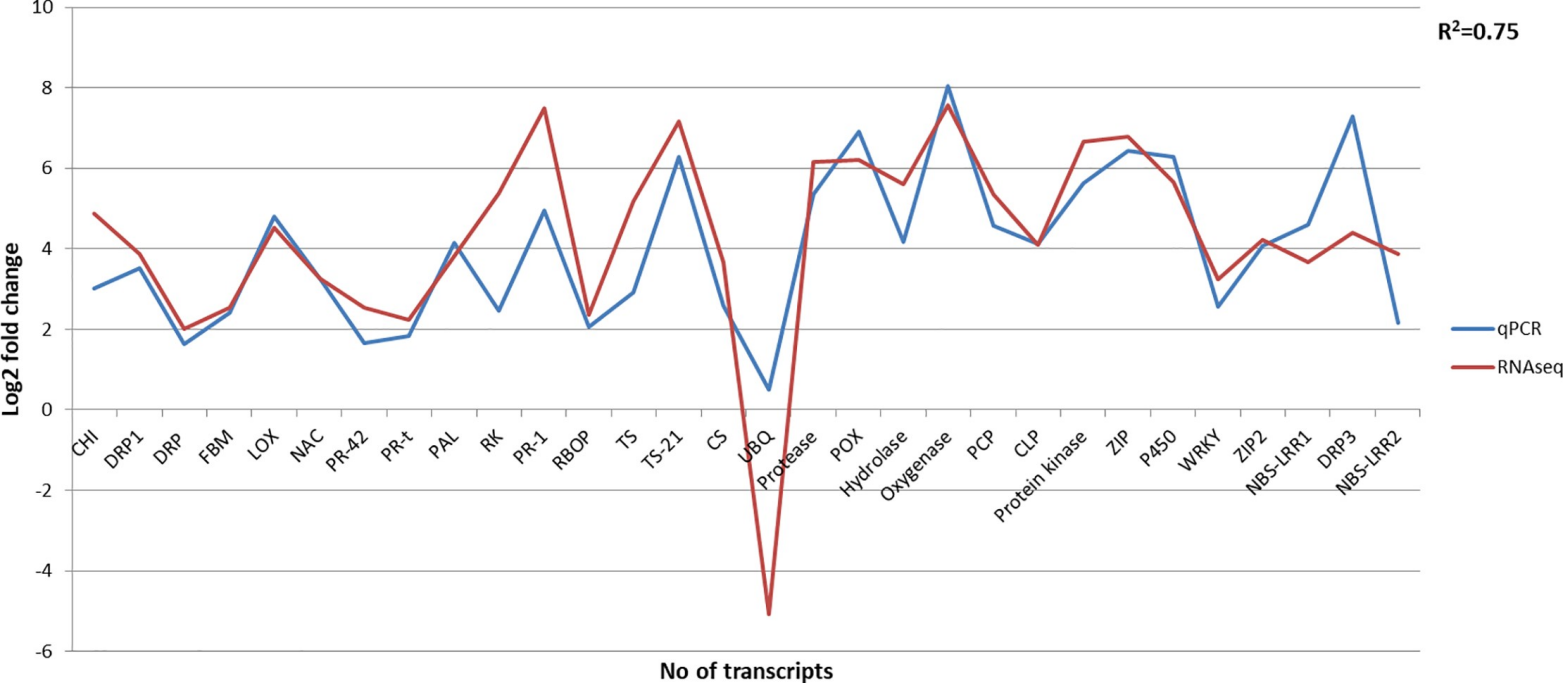
Correlation between differentially expressed transcripts from RNA-Seq and qPCR data for 30 genes.

Based on the functional classification of defense-related transcripts, 49 genes were selected for validation by qPCR technique. For endogenous control, the expression pattern of three endogenous genes such as Actin1, 18s rRNA, and tubulin was examined by RefFinder [31]; Actin1 ranked first in terms of stability and was then used for normalization of the relative expression of other target genes. Of 49 primers, 30 amplified a single amplicon, 10 amplified multiple amplicons, and nine primers could not amplify any product (S4 Table). Furthermore, the time course of expressions in 30 primers was observed in RI and SI samples and their respective controls (S3 Fig). The relative expression levels of the chitinase gene increased after 72 h post-inoculation in both resistant and susceptible samples; however, a gradual declining trend was observed at a subsequent time point of 96 h and 12 d post inoculation. At 12 d after inoculation, a three-fold increase in the expression of CHI was recorded in RI compared to SI sample; however, in the susceptible genotype, the expression of CHI was down-regulated with an increase in fungal infection. A similar pattern of expression was observed in disease-resistant protein (DRP), DRP1, Lipoxygenase (LOX), Receptor-like kinases (RLK), PR-thaumatin superfamily protein (PRT), pathogenesis-related protein (PR–1), terpene synthase–21 (TS–21), Calmodulin-like protein (CLP), WRKY transcription factor 65 like WRKY protein, and zinc-finger protein 2 (ZIP2). The expressions of respiratory burst oxidase protein (ROX), chalcone synthase (CS), and DRP3 were found more in the susceptible genotype than the resistant genotype. A constant increase in the expression of ROX was observed in the SI sample, whereas in the RI sample, it increased between 0 h to 72 h and then declined at 96 h after inoculation. A 2.2 fold increase in the expression of ROX was recorded in the SI sample compared to the RI sample at 12 d post-inoculation. A comparison of gene expression levels between RNA-Seq data and qPCR results was also carried out, and a positive correlation (R^2^ = 0.75) was recorded between differentially expressed transcripts from RNA-Seq and qPCR data for 30 genes (Fig 5).

## Discussion

### RNA-Seq data comparison

The present investigation was aimed to identify the genes imparting defense in the peanut plant against *S*. *rolfsii*. For artificial infection, the fungal hyphae (multiplied in sorghum grain) was used to inoculate the main stem of 70 days old plants; while mycelia agar plugs were used by Jogi et al. [22] to inoculated 50 days old plants. Multiple mycelial agar plugs are generally required to develop sufficient infection which also gets dried in very short period of time [22]. On contrary, the sorghum grain inoculum technique was reported more suitable for screening of stem rot resistance in groundnut under glasshouse conditions [5, 11] as the grains provide adequate nutrition to the fungus for longer period which helps in the development of uniform mycelia around the stem.

While carrying out disease progression analysis at different time points, disease symptoms appearing 12 days post-inoculation (dpi) were recorded in resistant genotype. Plants from both genotypes were inoculated, and at 12 dpi RNA-Seq was carried out for four samples including RI, SI, RC, and SC, from which 290 million reads were generated. Jogi et al. [22] studied the transcriptomic changes in peanut in response to *S*. *rolfsii* infection using a small number of reads (2 million). The comparison helped reduce the number of false-positive and false negative results, and 4514 transcripts could be identified.

The cultivated peanut (*Arachis hypogaea*) is an allotetraploid species having an AABB genomic constitution, with *Arachis duranensis* and *Arachis ipaensis* as the two most likely progenitors, which have supposedly contributed the A and B genomes, respectively [34–36]. The large, complex and polyploid genome of peanut has made it challenging to identify the expression patterns of homologous genes. Recently, the genome sequences of cultivated groundnut were released [37]. In the present study, genome sequences of two progenitor species of peanut, *Arachis duranensis* and *Arachis ipaensis*, were used for detailed analysis [36]. A total of 251 million clean reads were aligned to the genomic sequences of both *A*. *duranensis* and *A*. *ipaensis*. The average mapping rate of reads to the reference genome was nearly 78% per library, which was higher than that average mapping rate of reads of (62.7%) in transcriptome study, reported by Peng et al. [38].

### The defense interaction between peanut and *S*. *rolfsii* during infection

Plant defense responses get activated either upon pathogen recognition or by alterations of host cell structures/functions by the pathogen. The broad host range necrotrophs such as *S*. *rolfsii* can produce diverse groups of pathogen-associated molecular patterns (PAMPs), which are the structural component of the pathogen cell-wall or some macromolecules, activating PAMPs triggered immunity (PTI) [39]. Pathogen-derived PAMPs are directly perceived by cell-surface receptors, called pattern recognition receptors (PRRs).

In this study, 22 different types of RLK genes, including various LRR-RLK, serine/threonine protein kinases, cysteine-rich RLK, and calmodulin-binding receptor-like cytoplasmic kinase, were found to be stimulated in the inoculated resistant genotype. LRR-RLKs are known to be associated with the cell membrane, thereby generating various defense-related signaling cascades [40]. Furthermore, fungal PAMPs like endopolygalacturonase can be perceived by LRR-containing RLKs, and its downstream activation is known to induce the necrotic reaction in *Arabidopsis* [41].

In addition, RLPk–1, RLPK–2, RLPK–4, and cysteine-rich RLK were highly up-regulated in resistant genotype compared to susceptible genotype after *S*. *rolfsii* infection. Similarly, the elicitor SCFE1 was identified in *S*. *sclerotiorum* and perceived by receptor-like protein 30 (RLP30), and mutants of RLP30 were found to be more susceptible than WT plants [42]. Thus, RLPK seems to be associated with the recognition of specific elicitors of defense against *S*. *rolfsii in* peanut.

Damage-associated molecular patterns (DAMPs) are the elicitor molecules, which are released from the host cells during pathogen attack and evoke a quite similar response as PAMPs. Oligogalacturonides, peptides, and cutin monomers are the most characterized DAMPs, which elicit a wide range of defense responses in plants via cell wall-associated receptor kinases [40]. Moreover, the induction of PRRs is the first step in the activation of PTI [43]. Thus, the up-regulation of RLKs in the resistant peanut genotype seems to play a critical role in triggering downstream defense responses that involve complex signaling cascades against *S*. *rolfsii* infection.

### The immune interaction between peanut and *S*. *rolfsii* during infection

Although the induction of PTI is weak, the immune response against pathogen attack covers a broader spectrum. In contrast, effector-triggered immunity (ETI) can elicit a rapid and robust hypersensitive response (H) [44]. ETI response to the necrotrophic pathogen is rare. Excepting the *Arabidopsis RLM3*, a single dominant gene coding for TIR-NBS protein domains [45], no other R-gene that confer resistance to the necrotrophic pathogen, has yet been identified [46]. In the present study, 34 types of differentially regulated R-genes, such as disease resistance proteins (TIR-NBS-LRR class), dirigent proteins, CC-NBS-LRR, LRR, and NB-ARC domain, were identified in the resistance genotype in response to *S*. *rolfsii* infection. Although the functions of these genes in response to *S*. *rolfsii* infection are still unknown, it is suggested that they may have an effectors-specific immune response. However, further studies are required to identify the complex R genes conferring resistance by preventing interaction between peanut and stem rot fungi.

The immune responses in host plants are known to be triggered by signaling molecules such as mitogen-activated protein kinases (MAPKs), calcium-dependent protein kinases (CDPKs), and calcium-calmodulin binding proteins [47]. Likewise, calcium-dependent protein kinases, which might produce signaling cascades upon stimulation of *S*. *rolfsii*, were also found to be differentially regulated. The pathogen, triggering signaling cascades, directly targets and regulates the expression of several TFs, which play a vital role in generating downstream immune responses. Similarly, various classes of TFs such as WRKY, Zinc finger protein, C_2_H_2_ zinc finger, Ring-H_2_ finger protein, C_3_HC_4_-type zinc finger, bHLH, NAC, and MYB, were found to be differentially up-regulated in the resistant genotype compared to the susceptible genotype. Furthermore, the overexpression of *BnWRKY33* in *B*. *Napus* and *AtWRKY28* and *AtWRKY75* in *Arabidopsis* significantly enhances resistance against *S*. *sclerotiorum* infection [45, 48].

### Downstream gene expression in response to PAMP Triggered Immunity (PTI) and Effector-Triggered Immunity (ETI)

The downstream activation of PTI and ETI results in the activation of hormone-regulated signaling pathways, the backbone of the plant immune system [49]. It is well known that salicylic acid (SA) and jasmonic acid (JA) are the two major defense hormones; SA can regulate defense against biotrophic and hemibiotrophic pathogens, while JA and ethylene (ET)-dependent defense responses act against insect herbivores and necrotrophic pathogens [20, 50, 51]. The genes associated with the JA and ET signaling pathways were found to be up-regulated in peanut upon infection with *S*. *rolfsii*. Similarly, in *B*. *napus*, the ethylene branch of the JA-signaling pathway was found to be up-regulated, while the MYC TF-regulated JA responses in the MYC branch were down-regulated upon infection with *S*. *sclerotiorum* [20, 52–54]. A few studies have also emphasized the probable positive role of SA against necrotrophic infection in plants [54, 55]. However, no evidence of the SA-dependent signaling pathway operating against the infection by *S*. *rolfsii* in peanut was found. Further studies are required to establish the crosstalk between JA/ET and SA-dependent signaling pathways. Recent studies on the function of *Arabidopsis* receptor-like cytoplasmic kinase BIK1 in PTI to the necrotrophic fungus *Botrytis cinerea* demonstrate that BIK1 acts as a central regulatory element, which integrates multiple signals of PAMP, DAMP, ethylene (ET), and brassinosteroids (BRs) from surface-localized receptors to activate downstream defense responses [40]. Upon PAMP perception, BIK1 directly phosphorylates the respiratory burst oxidase homolog protein D (RBOHD) and induces the PAMP-mediated ROS burst and antibacterial immunity [56]. The signaling cascades and TFs impart resistance to *S*. *rolfsii* by the production of antimicrobial compounds such as PR proteins, defense-related proteins, antioxidative enzymes, and secondary metabolites, along with the fortification of the cell wall [57]. This study also revealed the high up-regulation of PR-proteins such as PR–1, PR–3, pathogenesis-related thaumatin superfamily protein, defensin related, thioredoxin, peroxidase, and chitinase in the resistant genotype compared to the susceptible genotype. Furthermore, PR proteins are activated at the site of infection and impart systemic acquired resistance (SAR), which, in turn, combats further fungal infection [22].

PR–3 type proteins or endochitinases are also induced upon pathogen attack and can be correlated with host resistance [58]. Similarly, upon infection by *S*. *rolfsii*, an upward trend was observed in the expression of the chitinase with an increase in fungal infection in both genotypes. However, the level of expression of the chitinase was much higher in the resistant genotype compared to the susceptible genotype. Similarly, two chitinase PR-3 and PR-4 were found to be up-regulated in resistant genotype when compared to the susceptible genotype [22]. Transient expression assay of the endochitinase gene in *B*. *napus* also revealed an increase in the resistance to *S*. *sclerotiorum* infection [59]. The increasing expression of inducible protein thaumatin-like proteins (PR-5) upon *S*. *rolfsii* attack was also reported in the previous study [22].

During infection, plant activates not only its defense-related genes but also genes involved in secondary metabolites production [60]. Similarly, the genes involved in the biosynthesis of flavonoids, phenylpropanoid, and terpenoids were found to be significantly up-regulated in the resistant genotype upon infection by *S*. *sclerotiorum*. The fungal pathogen is known to secret toxic compounds like oxalic acid, pectic enzymes and cellulase to destroy the host cell wall. Thus, the induction of glutathione reductase, polyamide oxidase and pectin esterase inhibitor in resistant genotype compared to susceptible genotype seems causing detoxification of such compounds as also reported in previous study [22].

It has been observed that genes associated with programmed cell death (PCD) were suppressed in the resistant genotype and up-regulated in the susceptible genotype. It is well documented that PCD induces necrosis in the plant, which in turn facilitates the growth of necrotrophic fungi by providing the substrate [44]. Similarly, a significant improvement of the growth of *Mycosphaerella graminicola* was observed in wheat after the activation of PCD [61]. Furthermore, susceptibility was induced by PCD in the susceptible genotype among tomato and *Verticillium dahlia*, whereas the genes associated with foliar necrosis and PCD, were found to be suppressed in the resistant genotype [62, 63].

### Candidate genes identified the main-effect QTLs region

Recently, Dodia et al. [5] have identified several main-effect and epistatic QTLs imparting resistance against *S*. *rolfsii* infection on the B genome through the genotyping-by-sequencing (GBS) approach. The relative position of three main-effect QTLs was correlated with the expression pattern of transcripts in the present study (S3 Table). The QTL region had several disease-responsive genes, such as kinase and peroxidase superfamily protein (B04). The WRKY transcription factor (TF on B06) may have a role in the large-scale transcriptional reprogramming through alteration of JA- and SA-dependent defense responses [64, 65]. Similarly, cinnamyl alcohol dehydrogenase (CAD on B10) playing a role in fungal resistance, was also found to be highly up-regulated in the resistant genotype compared to the susceptible genotype. Likewise, Rong et al. [66] have also reported over-expression of the *TaCAD12* gene imparting resistance to *Rhizoctonia cerealis* in wheat. The CAP superfamily protein (PR–1 on B10) seems to play an important role in imparting resistance through the up-regulation in the resistant genotype compared to the susceptible genotype. Breen et al. [67] also suggested multiple roles of PR–1 including antimicrobial activity and defense signal amplification upon effector recognition during pathogen attack. Moreover, Dodia et al. [5] reported six potential isoforms of NBS-LRR proteins imparting resistance to the stem rot in peanut.

## Conclusion

Fungal plant pathogens cause nearly 10% losses of the overall agriculture production, still are escalated after the growing crops get infected in the field [68]. The transcriptome profiling of stem rot in peanut has revealed the complex, massive, and coordinated changes in the genetic network, which has not only provided an insight into defense mechanism but also opened a novel perspective on the molecular mechanisms, leading to resistance to *S*. *rolfsii* in peanut. The results of the present study revealed that RLKs play a crucial role in the perception of the pathogen through a downstream resistance mechanism. Furthermore, upon the perception of the pathogen, the JA-mediated defense pathway gets activated, thereby inducing systemic resistance. In addition to RLKs, some R-genes such as TIR-NBS-LRR, dirigent proteins, CC-NBS-LRR, LRR, and NB-ARC domain protein, were also found to be differentially regulated; however, the role that most R-genes play is yet to be established. The roles of WRKY transcription factors, zinc finger proteins, lipoxygenase, terpene synthase, CAP protein superfamily (PR–1), and endochitinase genes, in imparting resistance to the stem rot are yet to be discovered for the first time. The information generated from in-depth transcriptomic studies can be useful for the identification of functional markers linked to stem rot resistance loci and also for designing better breeding strategies to combat this chronic disease.

## Supporting information

S1 Fig Seventy days old peanut plants where, A: NRCG-CS85 (resistant), B: TG37A (susceptible) inoculated with *S. rolfsii* at different time course; C and D: *S. rolfsii* infected peanut stem sections at 12 DAI in resistant and susceptible genotypes, respectively; E and F: Transverse section of stem showing compactness of tissue and cuticle thickness in resistant and susceptible genotypes, respectively.
(TIF)

S2 Fig Venn diagram representing the proportion of differentially expressed transcripts for the four sample comparisons.
(TIF)

S3 Fig Time course of log2 fold genes expression analysis through qPCR of resistant and susceptible genotypes against *S. rolfsii* infection.
(TIF)

S1 Table. List of differentially expressed Receptor-Like Kinases (RLKs) transcripts identified in RI_vs_RC comparison (XLSX)

S2 Table. List of differentially expressed resistance genes (R-genes) transcripts identified in RI_vs_RC comparison (XLSX)

S3 Table. Summary of stem rot resistance QTL underlying differentially expressed transcripts in four sample comparison (XLSX)

S4 Table. List of qPCR primer pairs used to validate expression pattern of transcriptome data (XLSX)
