# Supplementary figures and images for: Unraveling the mechanisms of resistance to Sclerotium rolfsii in peanut (Arachis hypogaea L.) using comparative RNA-Seq analysis of resistant and susceptible genotypes
Source: PLoS One. 2020 Aug 3;15(8):e0236823. doi: 10.1371/journal.pone.0236823 (PMC7398544; doi:10.1371/journal.pone.0236823)

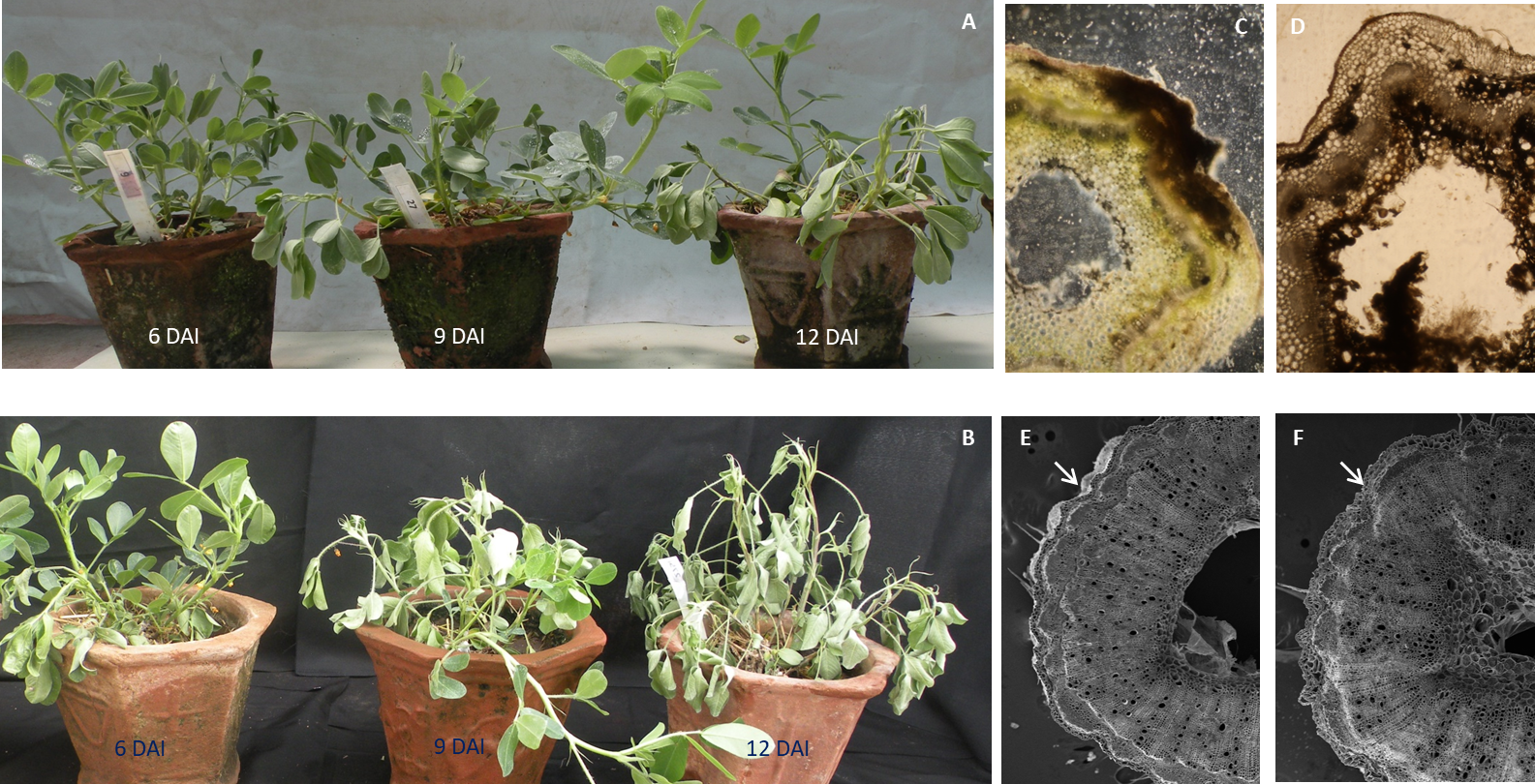

Supplement: S1 Fig — Seventy days old peanut plants where, A: NRCG-CS85 (resistant), B: TG37A (susceptible) inoculated with S. rolfsii at different time course; C and D: S. rolfsii infected peanut stem sections at 12 DAI in resistant and susceptible genotypes, respectively; E and F: Transverse section of stem showing compactness of tissue and cuticle thickness in resistant and susceptible genotypes, respectively. (TIF) [file pone.0236823.s001.tif]

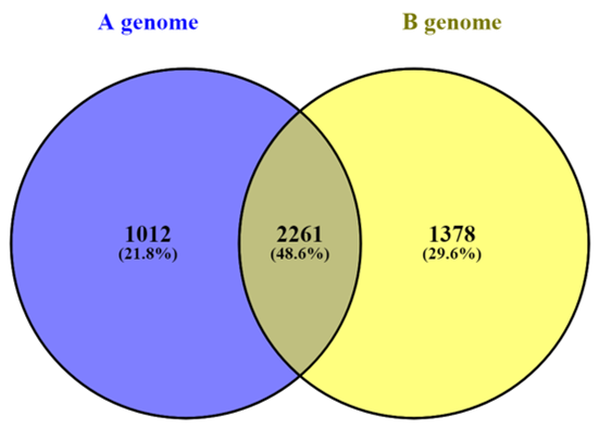

Supplement: S2 Fig — Venn diagram representing the proportion of differentially expressed transcripts for the four sample comparisons. (TIF) [file pone.0236823.s002.tif]

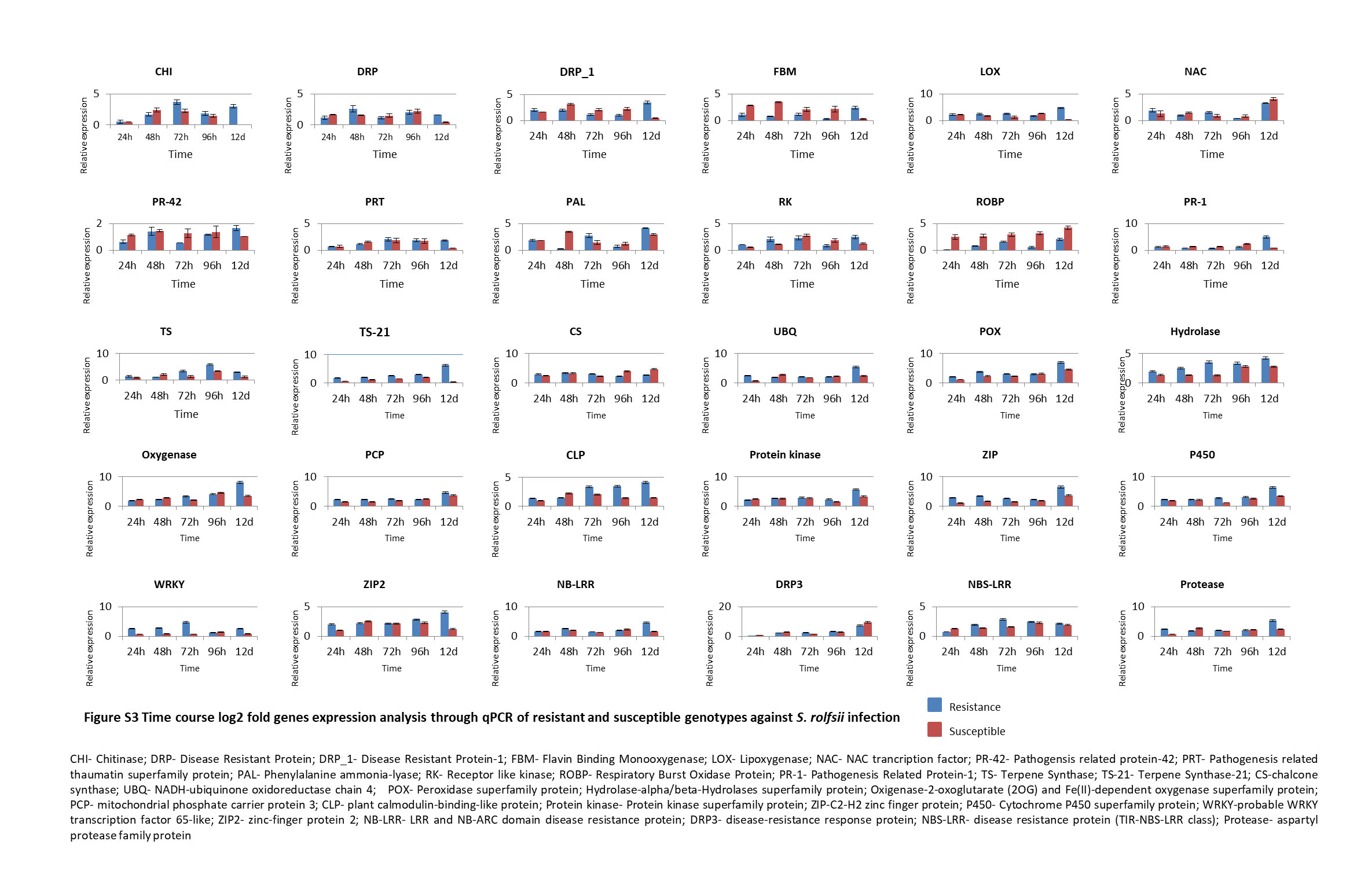

Supplement: S3 Fig — Time course of log2 fold genes expression analysis through qPCR of resistant and susceptible genotypes against S. rolfsii infection. (TIF) [file pone.0236823.s003.tif]
